# Processing and sectioning of organ donor spinal cord tissue for electrophysiology on acute human spinal cord slices

**DOI:** 10.1093/braincomms/fcag157

**Published:** 2026-04-29

**Authors:** Annemarie Dedek, Eder Gambeta, Raksha Shriraam, Emine Topcu, Jeff S McDermott, Jeffrey L Krajewski, Eve C Tsai, Michael E Hildebrand

**Affiliations:** Department of Neuroscience, Carleton University, Ottawa, ON, Canada K1S 5B6; Neuroscience Program, Ottawa Hospital Research Institute, Ottawa, ON, Canada K1Y 4M9; School of Pharmacy, University of Waterloo, Kitchener, ON, Canada N2G 1C5; Department of Neuroscience, Carleton University, Ottawa, ON, Canada K1S 5B6; Neuroscience Program, Ottawa Hospital Research Institute, Ottawa, ON, Canada K1Y 4M9; Neuroscience Program, Ottawa Hospital Research Institute, Ottawa, ON, Canada K1Y 4M9; Department of Neuroscience, Carleton University, Ottawa, ON, Canada K1S 5B6; Lilly Research Laboratories, Indianapolis, IN 46225, USA; Lilly Research Laboratories, Indianapolis, IN 46225, USA; Neuroscience Program, Ottawa Hospital Research Institute, Ottawa, ON, Canada K1Y 4M9; Brain and Mind Research Institute, University of Ottawa, Ottawa, ON, Canada K1N 6N5; Division of Neurosurgery, Department of Surgery, The Ottawa Hospital, Ottawa, ON, Canada K1Y 4E9; Department of Neuroscience, Carleton University, Ottawa, ON, Canada K1S 5B6; Neuroscience Program, Ottawa Hospital Research Institute, Ottawa, ON, Canada K1Y 4M9

**Keywords:** human, spinal cord, electrophysiology, patch-clamp, multielectrode array

## Abstract

Acute spinal cord slice electrophysiology is a powerful technique used in preclinical basic science research to investigate sensory and motor neuron function and pathophysiology. A major barrier that stands between implementing these findings into effective clinical treatments is the translational gap between rodent models and human patients. To date, no methods or protocols describe how to prepare viable human spinal cord slices for acute electrophysiological recordings. To bridge this translational divide, we describe here a protocol for the extraction of spinal cord tissue from consenting human organ donors and the preparation and sectioning of this tissue for acute spinal cord slice electrophysiology. With the collaboration of a transplant service and licensed surgeon, tissue can be extracted in 30–50 min. Acute spinal cord slices can then be prepared in the laboratory by trained graduate students in 2.5–5 h, depending on the amount of tissue and scope of experiments. Using a viability stain to confirm that spinal slices are of sufficient quality to proceed, slices can then be used for either patch-clamp recordings to study the excitability of individual neurons or for high-density multielectrode array recordings to study intact sensory circuits. Slices remain viable for 4–8 h, providing ample time for investigating synaptic and circuit-level signalling dynamics, including the use of pharmacological agents to probe the roles of specific molecular targets. The approaches described here can be implemented to improve translational physiological research and as a human tissue-based preclinical drug target identification and validation assay.

## Teaser

Dedek *et al*. present a protocol that enables stable electrophysiological recordings from healthy human spinal cord slices. Their findings validate this preparation for studying human spinal neurons and circuits and identify opportunities for cross-species comparisons and preclinical testing assays. These new approach methodologies move fundamental science towards human-specific clinical applications.

## Graphical Abstract

**Graphical Abstract:**
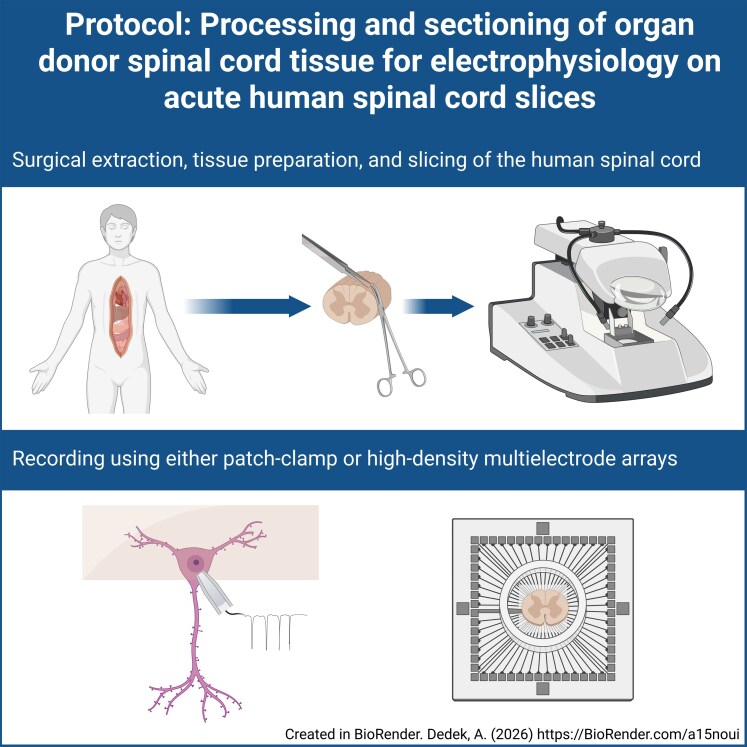

## Introduction

Studying the molecular determinants of sensory and motor processing in rodent preclinical models is a key first step towards the identification of new treatment approaches for diseases of the spinal cord. Preclinical human tissue assays are emerging to bridge the translational divide between these rodent models and target human populations, with a protocol for culturing human peripheral sensory neurons^1^ leading to transformational advances in understanding and targeting peripheral mechanisms of pain. Within a decade of using this published protocol,^1^ researchers across the globe have identified species differences in molecular determinants of sensory neuron physiology that are being leveraged towards new clinical treatments.^2-7^ These human peripheral neuron programmes reinforce a shift in both fundamental science and industrial drug development towards more predictive human tissue preclinical assays.^8,9^

The spinal cord is a central integrative hub that not only processes incoming sensory information but also generates and coordinates motor output, enabling reflexes, posture and voluntary movement through its networks.^10-20^ Molecular and synaptic changes within these dorsal and ventral circuits, including alterations in receptor expression, inhibitory–excitatory balance, glial signalling and intrinsic neuronal excitability, can profoundly reshape spinal output, contributing to diverse pathological states such as chronic pain, itch, spasticity, motor weakness and hyperreflexia. Many molecular targets have been identified as avenues for therapeutic development,^21-29^ but there has been little success in clinical translation to date.^21,30-34^

Although most fundamental spinal cord research has relied on rodents, the use of human spinal cord (hSC) physiology assays^35-37^ has opened the door to addressing these critical translational questions, similar to that done for human peripheral sensory neurons. These studies have allowed the comparison of receptor expression^38,39^ and neuronal biophysical properties^35^ across sex and species under non-pathological conditions, with many features being conserved across species and sex, but also some clinically relevant differences in receptor expression and function between rodents and humans.^35^ When characterizing signalling pathways in an *ex vivo* human tissue model of chronic pain, we uncovered that molecular determinants of pathophysiology are conserved across species^36,37^ but differ across sex.^37^ Highly viable tissue not used for electrophysiology can also be used to investigate transcriptomic profiles across species,^40-42^ as well as to compare the dorsal horn expression of a candidate therapeutic target, such as KCC2, between healthy donors and those with a history of pain or long-term opioid use.^43^

Despite the explosion of human sensory neuron research,^1,3,4,6,7,44-56^ there is a lack of physiology studies using hSC tissue. Electrophysiological approaches such as whole-cell patch-clamp and high-density multi-electrode arrays (hdMEAs) provide complementary, high-resolution readouts of human spinal neuron function in acute slice preparations. Patch-clamp enables direct measurement of synaptic currents, ion channel activity and intrinsic membrane properties at single-cell resolution, while hdMEAs allow simultaneous monitoring of network-level spiking across thousands of channels. Despite their central role in rodent spinal cord research, these *ex vivo* recording approaches have rarely been applied to hSC tissue due to challenges in access, preparation and cellular viability.

A particularly challenging technique to incorporate into hSC assays is acute slice electrophysiology, which is widely used in both rodent physiological and pharmacological studies to examine synaptic transmission, ion channel activity, membrane potential changes and network activity. The foundation of our understanding of neuronal physiology is based on animal models, and until the development of this assay, cross-species comparisons directly assessing baseline neuronal physiology within spinal circuitry have not been possible.^35^ Furthermore, conducting experiments using hSC tissue is an opportunity to validate pathophysiological pathways and molecular targets identified using rodent models.^36,37^

These functional outputs also provide opportunities to identify druggable targets and to test the efficacy of candidate drugs in human preclinical assays. Unlike the human sensory neurons described above, physiological studies on human CNS tissue have been mainly restricted to extracted pathological tissue in patients with epilepsy or brain tumors^57-59^ and are limited by the typically compromised cellular viability within the tissue that is collected. Here, we detail a protocol that can be used to prepare high-quality acute hSC slices collected from organ donors for either patch-clamp or high-density multi-electrode array (hdMEA) electrophysiological recordings on healthy dorsal horn neurons. Using this protocol on highly viable human donor tissue, researchers can cautiously plan to collect 1–2 neurons per donor for patch-clamp experiments and 4–5 slice recordings with pharmacology using an hdMEA. In addition, tissue that is not used for electrophysiology can be flash-frozen or fixed for use in parallel genetic, biochemical or immunohistochemical experiments.

## Development of the protocol

Building off of previous work on human sensory neurons^1^ and a protocol for culturing neural stem/progenitor cells (NSPCs) from the central canal of the human spinal cord,^60^ we have developed an assay for the preparation of viable hSC slices for electrophysiological recording. Similar to previous studies, this protocol employs an anterior surgical extraction approach, which provides the benefit of not requiring any additional incisions to be made to the skin of the organ donor, as well as not requiring the donor to be moved following organ procurement.^1,60^ This not only reduces the postmortem interval for spinal tissue collection and reduces the number of procedures requiring institutional review board approval, but also does not interfere with funeral arrangements for the donor. We then integrated elements such as protective tissue preparation solutions and tissue sectioning parameters from our previously refined and published rodent methodology, which consistently produces high-quality slices for electrophysiology in adult rat spinal cord tissue,^35-37,61-63^ which is also technically challenging. Finally, we made several important optimizations from the above-mentioned human and rodent protocols to account for the size of the tissue, its structural integrity and anatomical differences between species. The final procedures described here have resulted in an assay that reliably produces slices for electrophysiological examination of the hSC. These procedures include a surgical extraction technique that minimizes trauma to the spinal cord and nerve roots, a microdissection technique for preparing the hSC for sectioning and a sectioning assay using a high-quality vibratome that ensures viability is preserved during the sectioning process. Finally, a modified recovery setup for the slices enables adequate oxygenation of the slices, with a way to track individual slices to ensure slices recover for the correct amount of time at each slice recovery stage. These studies have allowed for the comparison of both synaptic glutamatergic signalling and circuit-level activity between sex and species, thus opening the door to translational research and drug screening assays.^35,36^

## Applications of the method

Although the present study focused on intrinsic dorsal horn physiology, this assay can be readily extended to investigate additional spinal circuits. In future work, slices can be prepared to retain ventral horn regions, enabling direct recordings from human motoneurons and the study of motor circuit physiology. Prior work from our group has shown that morphologically and biologically intact motoneurons are preserved in adult human spinal tissue.^40^ Likewise, while dorsal and ventral roots were intentionally removed for the current experimental configuration, it is technically feasible to preserve dorsal roots during slicing to allow the stimulation of afferent-evoked excitatory and inhibitory synaptic responses. Incorporating root-evoked physiology and ventral horn recordings therefore represent important avenues for expanding the use of this protocol towards both sensory and motor circuit research.

Beyond electrophysiology, the highly viable tissue slices prepared using this assay can be used in many other scientific applications. Tissue can be immediately flash-frozen in the operating room (OR) and used for single-nucleus RNA sequencing.^40,41^ These datasets can also be directly compared across species using spinal cord cellular atlases, such as in mice^64^ and macaque.^65^ These cross-species atlases provide a framework for interpreting conserved and divergent molecular features of spinal cord neuron subtypes.

Additionally, mechanisms of pathological pain can be modelled *ex vivo* by incubating slices in growth factors, such as brain-derived neurotrophic factor (BDNF).^36,37^ Tissue can be fixed in paraformaldehyde and frozen for immunohistochemical analysis.^36–39, 66^ Finally, the tissue can be flash-frozen for biochemical techniques such as western blot and quantitative PCR.^36,37,67^ Thus, complementary approaches can be paired with the hSC slice assay described here to ensure maximal use of the precious tissue, as well as to allow for the probing of scientific questions using multiple techniques.

## Experimental design

**Figure 1:**
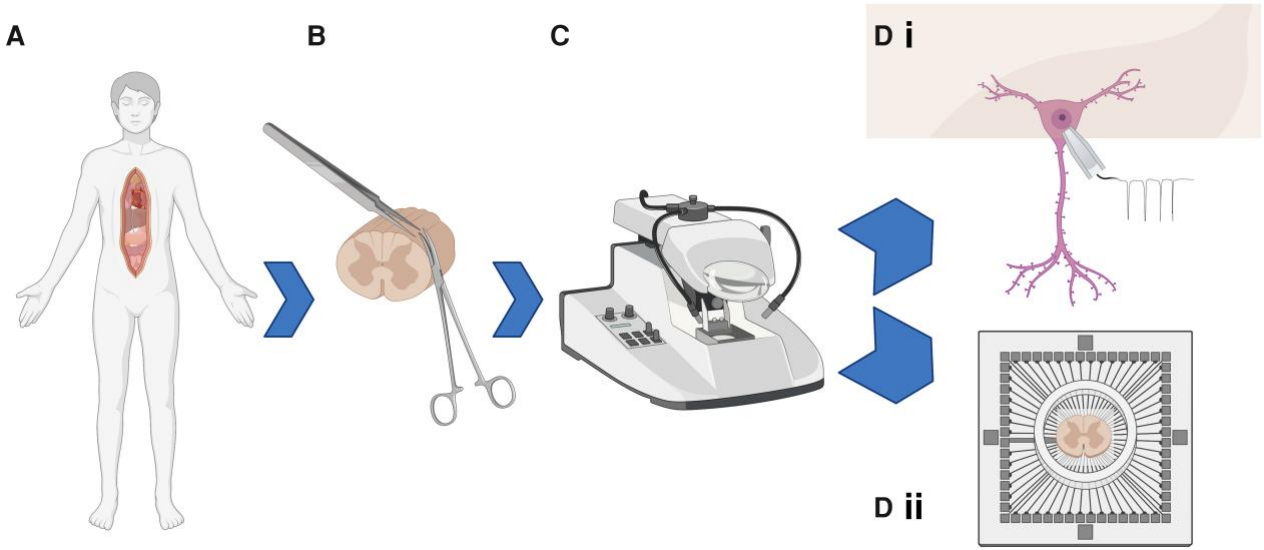
**Schematic of workflow for human tissue preparation for electrophysiology recording. (A**) Spinal cord tissue is first harvested from organ donors. (**B**) Tissue is then microdissected before (**C**) sectioning using a vibratome. (**D**) Spinal cord slices are then ready for electrophysiological analysis using either **D**i patch-clamp or **D**ii multielectrode array recording. Created in BioRender. Dedek, A. (2026) https://BioRender.com/viw2tcu.

Overview—the tissue preparation consists of four distinct stages: surgical extraction of hSC tissue, tissue microdissection, sectioning and recovery and use of slices for either patch-clamp or hdMEA recordings (Fig. 1). Following the removal of organs for transplant, hSC can be extracted from consenting donors in the operating room. hSC is collected into oxygenated protective aCSF solution containing kynurenate and is immediately transferred to the laboratory. Under constant bubbling with carbogen gas, hSC tissue is then microdissected to remove nerve roots and meninges and is then glued onto an agarose block and specimen plate, which are then placed into the buffer tray of a vibratome. At a slow speed and wide cutting amplitude, the hSC tissue is then sectioned into 500 µm slices. Slices are transferred to a custom recovery chamber in a water bath at 34°C for 40 min, after which slices are left to passively cool to room temperature for use in electrophysiological recordings.

Expertise needed to implement the protocol—At many institutions/hospitals, a licensed surgeon is required to oversee the tissue collection. Once tissue is collected, a graduate student or postdoc with experience in acute slice electrophysiology has the required skills to perform this protocol. To maximize the use of the acute tissue, it is suggested multiple team members share the tissue for parallel, concurrent electrophysiological experiments.

Access to human organ donor tissue—collection of hSC tissue is contingent on a collaboration with a transplant service or organ procurement organization (depending on the structure of the medical system where tissue is collected). After approaching the transplant service to establish a collaboration, appropriate regulatory approval and documentation are required, such as IRB approval, consent forms for family and establishing a protocol for the documentation required for consent. The specific protocol for access to the operating room, surgical tools available and the logistics for timing and notification will vary between institutions and must be established with the transplant service and institution. Since establishing our human tissue pipeline in 2017, we have received tissue on average 10 times/year, barring a temporary pause in tissue collection during the outbreak of COVID-19, at a trauma centre serving a population of ∼2 million people. At our institution, we are notified once a donor’s family consents to collection for our study. An operating room for organ collection is then scheduled, typically 24–48 h in advance.

## Limitations

As with any acute slice preparation, several factors may pose problems in the preparation of high-quality slices. Factors out of experimental control, such as the overall condition of the donor before undergoing organ harvest, as well as possible delays in extracting organs for transplant, may negatively affect the tissue. In some cases where few organs are collected, surgical extraction of the spinal cord may be difficult, due to lack of space in the body cavity to access the spinal column via an anterior approach. In such cases, it is likely that only 3–5 cm of hSC can successfully be harvested, whereas up to 30 cm can be extracted when all possible organs are harvested. We have found that tissue from all ages of donors can yield high-quality tissue, but donors who spent less time (<5 days) in intensive care before undergoing organ collection are more likely to produce high-quality tissue than those who were in ICU for an extended time. Furthermore, successful electrophysiological experiments can be performed from neurologic determination of death (NDD) and determination of cardiac death (DCD) donors; however, the shortened length of time between removal of supportive care and tissue collection in NDD cases yields more reliably high-quality tissue and a higher success rate for electrophysiological experiments. As is the case with all acute slice preparations, axotomy induces an inflammatory response and some cellular loss during the slice preparation is inevitable. TTC (2,3,5-triphenyltetrazolium chloride) staining provides a rapid and reliable assay of metabolic viability by labelling mitochondria capable of reducing TTC to a red formazan product.^68^ Viable tissue appears bright red, whereas damaged or non-viable regions remain white. The slice recovery process outlined here, paired with viability validation using TTC staining ensures tissue that is used for experiments is robust and of sufficient quality.

## Materials and methods

### Ethics approval

Ethics approval was obtained to collect and conduct experiments with human tissue by the Ottawa Health Science Network Research Ethics Board (Protocol ID No. 20150544–01 H) and the Carleton University Research Ethics Board B (Ethics Protocol No. 104836).

### Reagents

Tissue from human organ donors

- Extraction procedures and consent forms were approved by the Internal Review Board (IRB)
- Tissue should be extracted as soon as possible to minimize the postmortem interval; typically extract spinal cord tissue 1–3 h postmortem, immediately following surgical resection of donor organs for transplantation
- We have detected spikes/spontaneous activity with tissue extracted up to 4 h postmortem
- Take note of time of life-support withdrawal and cross-clamp time

- >96% Ethanol
- Carbogen gas (95% O_2_, 5% CO_2_)
- Deionized water
- TTC (Sigma-Aldrich, cat. no. T8877-10G)
  - Warning—flammable; can cause eye/skin irritation
- Agarose (Sigma-Aldrich, cat. no. A6013-100G)
- Instant adhesive (Loctite, part no. 46551)
  - Warning—Combustible liquid, causes eye irritation, may cause respiratory irritation and genetic defects
- Protective aCSF
  - Sucrose (Sigma-Aldrich, cat. no. S9378-1KG)
    - Warning—may form combustible dust concentrations in air
  - NaCl (Sigma-Aldrich, cat. no. 71380-1KG)
  - D-(+)-Glucose (Sigma-Aldrich, cat. no. G8270-1KG)
  - NaHCO_3_ (Sigma-Aldrich, cat. no. S5761-500G)
  - KCl (Fisher, cat. no. P217-500)
    - Warning—causes eye irritation; may cause respiratory tract irritation
  - NaH_2_PO_4_ (Sigma-Aldrich, cat. no. S0751-500G)
  - CaCl_2_ dihydrate (Fisher, cat. no. BP510-100)
    - Warning—causes serious eye irritation
  - MgSO_4_ anhydrous (Fisher, cat. no. M65-500)
  - Kynurenic acid (Sigma-Aldrich, cat. no. K3375-5G)
- Slice External Recording Solution
  - NaCl (Sigma-Aldrich, cat. no. 71380-1KG)
  - KCl (Fisher, cat. no. P217-500)
    - Warning—causes eye irritation; may cause respiratory tract irritation
  - NaHCO_3_ (Sigma-Aldrich, cat. no. S5761-500G)
  - NaH_2_PO_4_ (Sigma-Aldrich, cat. no. S0751-500G)
  - CaCl_2_ dihydrate (Fisher, cat. no. BP510-100)
    - Warning—causes serious eye irritation
  - MgCl_2_ hexahydrate (Fisher, cat. no. BP214-500)
    - Warning—irritating to eyes and respiratory system
  - D-(+)-Glucose (Sigma-Aldrich, cat. no. G8270-1KG)
- Internal Recording Solution
  - Gluconic acid (Fisher, CAS no. 526-95-4)
    - Warning—causes severe skin burns and eye damage
  - CsOH monohydrate (Fisher, CAS no. 35103-79-8)
    - Warning—causes severe skin burns and eye damage.
  - CsCl (Fisher, CAS no. 7647-17-8)
    - Warning—reproductive toxicity, suspected of damaging fertility or the unborn child
  - BAPTA, 1,2-bis 2-aminophenoxy ethane-*n*,*n*,*n*′,*n*′-tetraacetic acid (Thermo Fisher Scientific, CAS no. 85233-19-8)
  - HEPES (Fisher, cat no. BP310-500)
    - Warning—may cause respiratory irritation
  - Mg-ATP (Sigma-Aldrich, cat. no. A9187-1G)
    - Warning—may cause damage to organs
  - Na_2_-GTP (Sigma-Aldrich, cat. No. G8877-100MG)

### Equipment

Surgical Spinal Cord Extraction

- Orthopaedic mallet (Blacksmith Surgical, cat. no. BS-13-34011)
- Stryker System 7 Sternal Saw (not autoclavable, cat. no. 7207-000-000)
- 32 mm straight-tip bone osteotome (Blacksmith Surgical, BS-13-34329)
- DeBakey tissue forceps (Sklar Surgical Instruments, cat no. 52-5307)
- Straight mayo scissors (Sklar Surgical Instruments, cat no. 15-1555)
- Metzenbaum scissors (Sklar Surgical Instruments, cat no. 22-1507)
- Harrington-Mixter clamp (Sklar Surgical Instruments, cat no. 55-3012)
- Scalpel handle #3 (BS-01-10001) with no. 10 blades (Bard-Parker, cat. no. 371110)
- 50 ml Conical tubes (Greiner, item no. 210270)
- Container filled with ice (sufficient to accommodate 6 conical tubes)

Spinal Cord Microdissection

- Stereo microscope (Leica, M50)
- Dumont #5 straight-tip forceps (Fine Science Tools, item. no. 11251-10)
- Scalpel handle with no. 10 blades (Bard-Parker, cat. no. 371110)
- DeBakey tissue forceps (Sklar Surgical Instruments, cat no. 52-5307)
- Straight-edge Vannas microdissection scissors (World Precision Instruments, cat. no. 500086)
- 50 ml petri dishes (VWR)
- Styrofoam tray

Tissue Sectioning

- Vibratome with Vibrocheck (Leica, VT1200S)
- Water Bath (Fisher Scientific, Isotemp 2340)
- Custom glass dropper
- 1L Beaker
- 300 ml petri dish (VWR)
- 2× Custom slice holder, or commercially available large slice holder

Patch-Clamp Recordings

- Flaming/brown micropipette puller (Sutter Instrument, P-97)
- Microforge (Narishige, MF-83)
- Borosilicate glass capillary tubes (1.5/1.17 mm outer/inner diameter) (Sutter Instrument, item no. BF150-117-10)**
- Microscope Axio Examiner.A1 (Zeiss)
- Headstage (Molecular Devices, CV-7B)
- Amplifier (Molecular Devices, MultiClamp 700B)
- Digitizer (Molecular Devices, Digidata 1550)
- Infrared Microscopy Camera (Dage-MTI, IR-1000)
- Micromanipulator (Scientifica, PS-7500)
- Peristaltic pump (Fisher, CTP300)
- Tygon tubing, assorted sizing (Fisher, cat no. 14-179-110)
- Tissue anchor (Warner Instruments)
- Optical Table (ThorLabs)

High-Density Microelectrode Array (hdMEA) Recordings

- hdMEA system (3Brain, BioCAM X)
- Peristaltic pump (Fisher, CTP300)
- Tissue anchor (3Brain)
- Tygon tubing, assorted sizing (Fisher, cat no. 14-179-110)
- Blunt-fill 16G needles (Fisher, cat no. BD 305180)

Software

- BrainWave v.5 (3Brain)
- Clampfit v.11.2 (Molecular Devices)

*See equipment setup

**! Critical step

### Reagent setup

#### 5% Agarose

5% (w/v) solution in protective aCSF, heated to dissolve agarose and refrigerated to set in a petri dish. The depth of the agarose gel should be 1 cm.

#### Protective aCSF

To prepare 1L of protective aCSF, combine the reagents listed in Table 1 below in deionized water. Store at 4°C and use within 2 days. Immediately prior to use, allow to bubble with carbogen for a minimum of 20 min.

**Table 1:** Reagents for protective aCSF

Reagent	Final concentration (mM)	Molecular weight (g/mol)	1L
Sucrose	50	342.3	17.115 g
NaCl	92	58.44	5.376 g
Glucose	15	180.156	2.702 g
NaHCO_3_	26	84.007	2.184 g
KCl	5	74.5513	0.373 g
NaH_2_PO_4_	1.25	119.98	0.172 g
CaCl_2_	0.5	110.98	0.074 g
MgSO_4_	7	120.366	0.843 g
Kynurenic acid^a^	1^a^	189.17	0.039 g^a^

Freeze extra protective aCSF with kynurenate in an ice cube tray in advance of hSC experiments. To help keep the preparation cold during tissue extraction, microdissection and sectioning, ensure some protective aCSF ice is always visible at all stages up until slice recovery. ^a^Dissolve 0.039 g of kynurenic acid into 200 ml of protective aCSF the day of experiments.

#### Slice external recording solution (aCSF)

To prepare 1L of slice external recording solution, combine the reagents listed in Table 2 below in deionized water. Store at 4°C and use within 1 week. Immediately prior to use, allow to bubble with carbogen for a minimum of 20 min.

**Table 2:** Reagents for slice external recording solution (aCSF)

Reagent	Final concentration (mM)	Molecular weight (g/mol)	1L
NaCl	125	58.44	7.305 g
KCl	3	74.5513	0.224 g
NaHCO_3_	26	84.007	2.184 g
NaH_2_PO_4_	1.25	119.98	0.172 g
CaCl_2_	2	110.98	0.294 g
MgCl_2_	1	95.211	0.203 g
Glucose	20	180.156	3.603 g

#### Internal recording solution

To prepare 10 ml of internal recording solution, combine all reagents listed in Table 3 except for Mg-ATP and Na2-GTP in 9.5 ml of deionized water. Adjust the pH to 7.25 with CsOH and check the volume to ensure it amounts to 10 ml. Add Mg-ATP and Na2-GTP. 295 mOsm. Filter then freeze 1 ml aliquots and store at −20°C and use within 6 months. Although a caesium-based internal solution was used here to isolate synaptic currents, other internal formulations such as a potassium-based internal solution can be substituted for additional scientific objectives, such as studying intrinsic excitability.

**Table 3:** Reagents for internal recording solution

Reagent	Final concentration (mM)	Molecular weight (g/mol)	g/10 ml
Gluconic acid	105	196.16	0.206
CsOH	105	149.912	0.157
CsCl	17.5	168.36	0.029
BAPTA	10	380.35	0.038
HEPES	10	238.3012	0.024
Mg-ATP	2	529.47	0.0101
Na_2_-GTP	0.5	523.18	0.0026

### TTC staining solution

Prepare 10 ml of a 2% w/v solution of TTC in room temperature PROTECTIVE ACSF without kynurenate that has been bubbled with carbogen.

### Equipment setup

#### Sternal saw

Ensure the blade of the sternal saw is facing away from the handle, so that the saw cuts when pushing the saw away from you. Often this required turning the blade 180°.

#### Vibratome calibration

Securely attach the vibrating microtome blade and follow Vibrocheck calibration instructions as described in the manufacturer’s protocol. Ensure a new blade is installed and the system is calibrated. This calibration is critical to ensure that the vertical deflection of the blade is minimized, thus producing slices with viable cells on the surface of the slice. **

#### Custom glass dropper

Modify a standard glass Pasteur pipette by scoring the taper of the pipette with a diamond-tip glass cutter. Then break the tip off of the pipette and attach the bulb to the cut end. A glass dropper gives more precision when transferring slices between solutions and reservoirs, and tissue is less likely to attach to it.

#### Custom slice holder

Cut 1 inch polyvinyl chloride (PVC) piping, and using hot glue, assemble them together to form a 9-unit grid. Secure the plastic screen beneath the grid using the same adhesive (see Supplementary Fig. 1). Commercial options are also available.

#### Water bath for slice recovery

Place a 1L beaker filled with ∼400 ml PROTECTIVE ACSF (without kynurenate) in a water bath. Place the custom slice holder in the beaker; it will float just below the surface, ensuring slices are separated and immersed in PROTECTIVE ACSF, without sitting on the bottom of the beaker and risking having an area that is not exposed to fresh oxygenated solution. Heat the water bath so that the temperature of the PROTECTIVE ACSF is exactly 34°C. Bubble the PROTECTIVE ACSF continuously with carbogen.

#### Patch-clamp rig and hdMEA

Ensure all components of the equipment are on, aCSF solution is being bubbled with carbogen in the perfusion reserve, and recording software is open.

#### hdMEA chip preparation

If required based on manufacturer recommendations, 3 days prior to recordings fill the chip chamber with PBS to hydrate the chip. Immediately prior to use, clean the contact pads of the chip with 96% ethanol and allow time to dry. Rinse the reservoir once with ethanol and twice with distilled water.

#### Recording glass pipette preparation

To pull the patch pipette, mount the borosilicate glass capillary tube onto the puller as described in the manufacturer’s protocol. Using the microforge, gently fire-polish the glass patch-clamp pipettes, with a ∼1 μm tip opening and 6–12 MΩ resistance once filled with internal. Ensure the pipette has a tapered and conical structure to facilitate sealing and prevent plugging.

### Spinal cord extraction (timing—30–50 min)

#### CRITICAL

The amount of space within the visceral cavity to perform the following steps will vary depending on the organs harvested for donation, as well as the amount of visceral body fat of the donor. The procedure is made more difficult with fewer organs removed and/or if the donor has a large amount of visceral fat. In the case of limited access to the spinal column, extend as rostrally as possible. The dissection tools used below were made available for this extraction protocol in the operating room. Investigators should coordinate with their transplant service and hospital regarding whether they need to provide their own instruments.

#### ! CAUTION

Sterile operating room procedures must be followed for tissue extraction in the operating room. Exercise proper safety precautions (e.g. gloves, mask, eye protection, scrubs, surgical gown) when working with human tissues.

**Figure 2:**
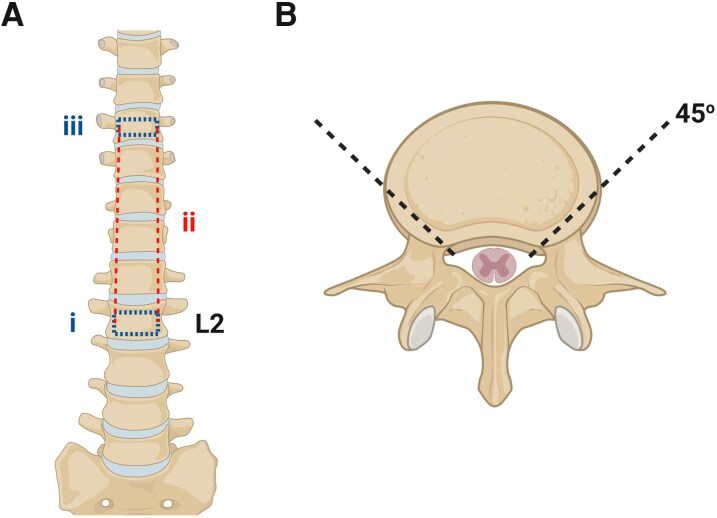
**Surgical approach for spinal cord extraction.** (**A**) i: Using a straight, wide osteotome and surgical mallet, the L2 vertebral body is transected, stopping at the spinal canal. ii: The two incised vertebral bodies are connected by an incision made using a sternal saw. iii: The most superior vertebral body that is accessible is transected to allow for the removal of the section of vertebral disk and expose the spinal canal. (**B**) A 45° angle must be used in ii, ensuring the sternal saw clears the vertebral bodies and without damaging the spinal cord beneath. Created in BioRender. Dedek, A. (2026) https://BioRender.com/5d2s0wc.

1. Immediately before leaving the laboratory space to go to the operating room, prepare several 50 ml conical tubes filled with bubbled 0–4°C protective aCSF containing kynurenate in a Styrofoam box filled with ice. Ensure that the conical tubes have screw-top lids, which are securely tightened to avoid dissipation of oxygen. The remaining protective aCSF should continue to be bubbled on ice in the laboratory space during the spinal cord extraction.
2. Using a surgical towel and retractor, contain the remaining organs to expose the spinal column.
3. Locate the sacral promontory. Then, count the lumbar vertebrae to identify L2.
4. Use an osteotome and mallet to make a transverse, wedge-shaped osteotomy through the L2 vertebral body (Fig. 2Ai). Ensure the wedge is sufficiently wide to expose the spinal canal and allow the footplate of the sternal saw to be placed inside the spinal canal, without penetrating the dura.
5. Mobilize all organs to one side, exposing as much length of the spinal column as possible on one lateral side. Insert the footplate of the sternal saw and angle the saw at 45° medially (Fig. 2B). Pushing the sternal saw away from you, cut through the vertebral bodies in a caudal to rostral direction as rostrally as the accessibility within the body cavity will allow (Fig. 2Aii). In cases where the heart and lungs are removed, this may be to the top of the surgical incision in the ribcage. If many organs remain, cut as high as possible. Then move the organs to the other side and repeat on the other side.

CRITICAL STEP

Once inserted into the spinal canal, it is critical to hold the footpad of the saw firmly against the anterior wall of the spinal canal to ensure the footpad does not damage the spinal cord below.

CRITICAL STEP

Do not change the angle of the sternal saw. Changing this angle may break the blade of the saw or result in difficulty retrieving the saw.

**? Troubleshooting** (Table 4)

6. Use a straight osteotome to detach the vertebral bodies, taking care not to perforate the dura or damage the spinal cord below (Fig. 2Aiii).
7. Using a closed Harrington-Mixter clamp to gently lift the thecal sac, transect the thecal sac with Mayo or Metzenbaum scissors at the L2 level. Holding only the dura (not any roots or the hSC), gently lift the thecal sac. Using Mayo or Metzenbaum scissors, transect the nerve roots and fascia that hold the thecal sac in the spinal canal. Once the exposed length of the thecal sac is freed, transect the rostral end of the exposed thecal sac. Transfer to a folded surgical towel for microdissection.

**Table 4:** Troubleshooting guide

Step	Problem	Possible reason	Solution
5	Sternal saw cannot span the vertebral bodies	Large vertebral body diameter	Use a straight osteotome to perform the procedure. This adjustment may be necessary, but significantly increases the time required for hSC extraction
12	Meninges easily peeling away from white matter beneath; appearance of ‘peach fuzz’ beneath meninges	Spinal cord is disintegrating and may not be viable for electrophysiology	Using a scalpel, remove a small section of the hSC and test for viability using TTC. If no red staining appears, do not proceed with slicing and recording
18	Slices are tearing or lack integrity	This could happen for a number of reasons—please match possible reason with correspondingly numbered solution. Tissue is of insufficient quality to slice Meninges are catching the blade and tearing tissue Glue has wicked in front of the blade and is obstructing the blade	The tissue may be damaged due to the circumstances surrounding death of the donor, or because of issues with the tissue extraction. Review extraction to ensure that no force was put on the hSC sample during extraction If another tissue sample is available, restart the procedure with a new hSC segment and ensure to carefully remove ALL roots and meninges If another hSC tissue sample is available, restart and ensure a minimal amount of glue is used to secure the hSC on the vibratome chuck
20	Slice is not changing colour to red following TTC staining	The sample is damaged, and no viable cells remain	Carefully examine all previous stages to determine if something could have damaged the hSC sample. If no reason is identified, it may be due to the circumstances surrounding the death of the donor
23	Cracks emerge in the hSC sample while it is mounted to the specimen plate	Damage during extraction or microdissection, incorrect sectioning parameters, required more agarose support	If tears or cracks appear in the hSC tissue sample while it is being sliced, increase the thickness of the slice to get past the damaged area. Slow down the cutting speed and ensure a cutting blade amplitude of 2.75 mm is being used. Ensure the tissue sample is being supported by the agarose block
25–27	No clear substantia gelatinosa, blebby or dark appearance of neurons under brightfield optics	The tissue may not be viable for patch-clamp recording	Repeat Step 20 (TTC staining) to check for slice viability with one of the remaining slices. If no colour change is observed, end experiments, as the tissue is no longer viable
25–31	No activity in recordings	Slices may no longer be viable	Repeat Step 20 (TTC staining) to check for slice viability with one of the remaining slices. If no colour change is observed, end experiments, as the tissue is no longer viable

CRITICAL STEP

Do not lift the dura more than 20° from the spinal canal, and do not put excess tension on the thecal sac. Lifting the thecal sac at too abrupt an angle will severely damage the grey matter of the hSC. It is better to carefully cut under and around the thecal sac without fully being able to see than to lift it too high.

8. On a surgical towel, use forceps to lift the dura off the spinal cord. Using Mayo scissors, quickly and gently cut from caudal to rostral, exposing the hSC.
9. Using forceps and a number 10 blade, section the hSC into 1.5 cm pieces and immediately place in prepared, ice-cold, pre-bubbled protective aCSF with kynurenate for transport to the laboratory.

CRITICAL STEP

Time sensitive. It is critical to get the extracted tissue into oxygenated protective solution as quickly as possible; it is critical that the number 10 blade is used gently. Unlike typical scalpel use, we recommend using the forceps to gently secure the hSC on either side, not putting any pressure on the hSC itself. Then, using only the weight of the number 10 blade and handle, slowly saw back and forth across the hSC. Using the typical scalpel technique of one swift incision creates excess shearing force and downward pressure and can be very damaging to the hSC.

TIP: We have found that using the conus terminalis/sacral spinal cord region for electrophysiological recordings is the most practical, as the smaller tissue diameter makes sectioning easier.

### Spinal cord microdissection (timing—30–60 min)

#### ! CAUTION

Follow proper safety precautions when working with and disposing of materials containing human tissues.

**Figure 3:**
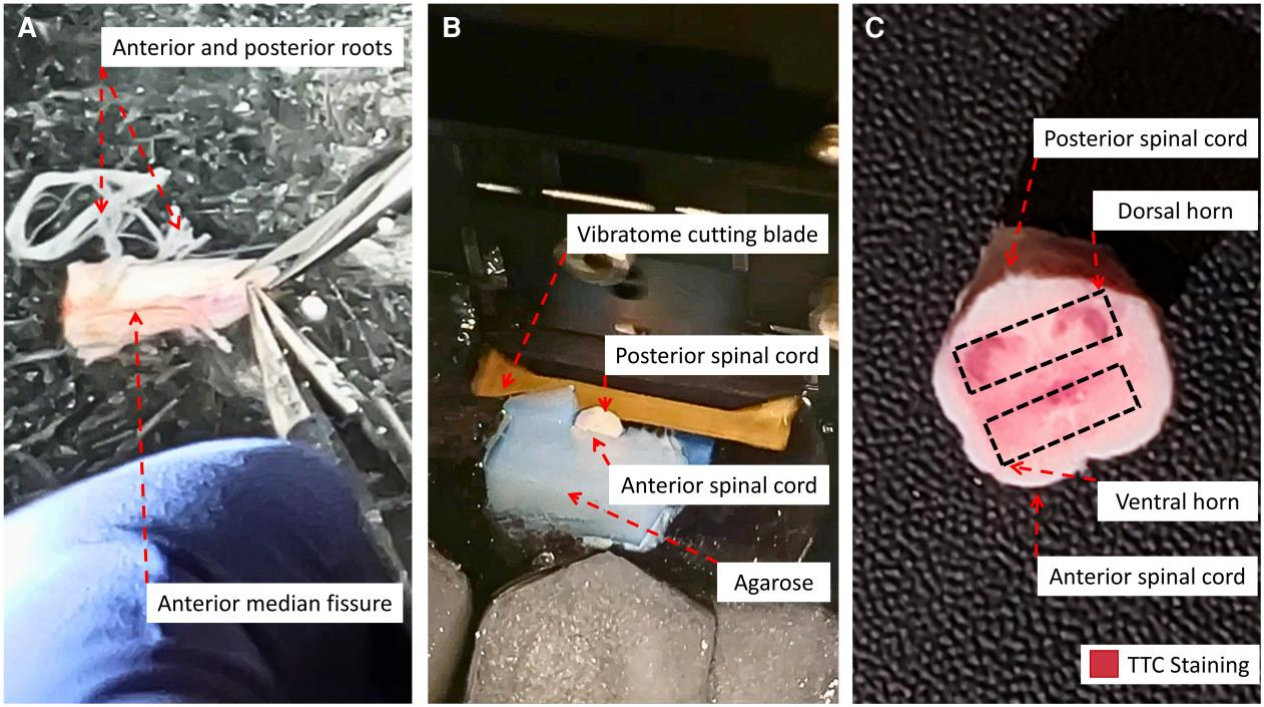
**Spinal cord preparation.** (**A**) Tissue microdissection: it is critical to remove all roots, followed by all meninges prior to sectioning. (**B**) Sectioning tissue on a vibratome, using blocks of agarose to support the spinal cord. The spinal cord is secured using glue on the anterior side for examination of the posterior horn (equivalent to the dorsal horn in rodents). (**C**) The first slice removed during sectioning is placed in a 2% 2,3,5-triphenyltetrazolium chloride (TTC) solution for 30 min as a readout of tissue viability before proceeding with experiments.

10. Place a piece of hSC in a 100 mm petri dish filled with ice-cold protective aCSF with kynurenate to fully submerge the hSC. Keep the petri dish on ice in a Styrofoam tray for the duration of cleaning and dissection. Bubble the protective aCSF with carbogen and continually add protective cutting solution ice cubes as they melt over time.
11. Using Dumont #5 straight-tip forceps and straight-edge Vannas microdissection scissors, gently remove the anterior and posterior roots. Begin by securing a root with the forceps, and with the scissors gently follow the root to the surface of the hSC. Gently, using as little force as possible, align the scissors so they are parallel to the surface of the hSC and cut to remove the root (Fig. 3A, Supplementary Video 1). Repeat for all roots.
12. Using Dumont #5 straight-tip forceps and straight-edge Vannas microdissection scissors, pinch the remaining meninges as superficially as possible and cut to remove a small portion of the meninges. Continue this process piece by piece until all the meninges are removed (Supplementary Video 1).

CRITICAL STEP

This step is painstaking and slow, but the utmost care should be taken to remove as much of the meninges as possible to ensure sectioning on the vibratome does not damage the slices. We have found that the more difficult the removal of the remaining meninges, the higher quality of hSC. If the pia and arachnoid layer easily peel away, or if there is excessive fraying of the underlying white matter, this may indicate the hSC is disintegrating and not viable for experiments. To fully remove the remaining meninges, it is often necessary to pinch and cut away a small amount of the superficial white matter. This does not impact the underlying grey matter, which is located more internally than in the rodent spinal cord.

13. Perform a visual inspection of the exposed area of the grey matter to ensure that there are no haematomas or other damage and that the edge of the tissue is straight and perpendicular to the length of the hSC. If the edge is not straight or there is damage, use DeBakey tissue forceps and a number 10 blade to remove tissue and straighten out the edge of the piece of hSC, as described in Step 9.

### Spinal cord sectioning and recovery (timing—2–4 h)

#### ! CAUTION

Follow proper safety precautions when working with and disposing of materials containing human tissues.

14. Using a razor blade, cut a piece of agarose that is barely longer and wider than your hSC segment (typically 12 mm × 14 mm). Ensure the agarose has ∼90°corners to make sure the slices will be straight. Glue one of the two smallest faces of the agarose block to a vibratome specimen plate using instant adhesive (Fig. 3B, Supplementary Video 1). Confirm that the angle created by the chuck and the agarose block is 90°.
15. Place a small dab of instant adhesive on the specimen holder directly in front of the agarose block. Place a thin line of adhesive on the edge of the agarose that faces the first dab of adhesive. With a folded kimwipe, spread the adhesive into a thin, even coat.

CRITICAL STEP!

Do not use excessive adhesive. Excess adhesive may wick around the hSC when solution is poured in and then may catch the vibratome blade, damaging the slices.

16. Using DeBakey forceps and the flat end of a scoopula, pick up the hSC segment by the posterior side if studying the posterior (dorsal in rodents) horn and by the anterior side of studying the anterior (ventral in rodents) horn. Gently touch the edge of the hSC segment to the edge of the petri dish to allow excess protective aCSF to flow off of the hSC segment. Gently place the hSC segment on the specimen holder and agarose block covered in adhesive, with the area of interest (anterior or posterior horns) facing out (Fig. 3B, Supplementary Video 1).
17. Immediately place the specimen holder with the attached hSC segment in the buffer tray of a vibratome, with the ice tray lined with ice to ensure it remains ice-cold throughout sectioning. Immediately immerse in ice-cold protective aCSF with kynurenate bubbled with carbogen, by pouring the solution gently into the side of the buffer tray until it reaches the height to completely submerse the spinal cord segment. Bubble with carbogen throughout sectioning and continually add protective cutting solution ice cubes to maintain a temperature of 0–4°C.
18. At a cutting speed of 0.01–0.02 mm/s and a horizontal blade amplitude of 2.75 mm, remove a thick (>500 μm) slice off the mounted hSC. This slice should be thick enough to create a level surface from which future 500μm slices can be cut. A number 10 scalpel blade may be required to free the slice from the agarose block (Supplementary Video 1).

**? Troubleshooting** (Table 4)

19. While this first slice is being sectioned, prepare the TTC solution.
20. Place the freshly sectioned thick hSC slice in a well of a 6-well plate, immersed in TTC solution. Place in a cell-culture incubator for 30 min. Continue with sectioning while the slice is incubating in TTC. After 30 min, check to see if a red colour change has occurred throughout the grey matter, indicating mitochondrial activity and slice viability^68^ (Fig. 3C). If the slice does not change colour to red, the tissue is not viable for electrophysiological recordings. Try once more with another slice, and if no colour change occurs once more, end the process here.

**? Troubleshooting** (Table 4)

21. After the first thick hSC slice, section the hSC at 500 µm. When the first 500 µm slice is ready, release it from the agarose block using a number 10 blade and use the custom dropper to transfer the slice to the prepared custom slice recovery chamber (Supplementary Fig. 1) in the heated water bath.
22. Set a running timer. Continue sectioning and placing slices sequentially in the slice recovery chamber. Forty minutes after the first slice was put in the slice recovery chamber, use a motorized pipettor to remove ∼150 ml of 34°C protective aCSF from the 1L beaker and place it in a 300 ml petri dish containing a second, smaller custom slice holder. Move the first slice from the first chamber in the second slice holder, and ensure this solution is bubbled with carbogen. This 300 ml petri dish will passively cool to room temperature in ∼30 min, after which the slice can be used for experiments.
23. Continue sectioning. After transferring the first slice out of the water bath slice recovery chamber, move one additional, sequential, slice from the water bath slice recovery chamber to the room temperature recovery using the custom dropper after each new slice is finished on the vibratome. Note the approximate time it takes for one slice to finish sectioning.

**? Troubleshooting** (Table 4)

24. Once you are finished sectioning on the vibratome, set a timer for the amount of time it took to complete sectioning a slice. When this timer is up, move the next-oldest slice from the water bath recovery to the room temperature recovery. Ensure slices remain at room temperature recovery for 30 min before starting experiments.

Note: If you have multiple team members working, you can begin electrophysiology experiments while one team member continues sectioning and managing slice recovery.

### Electrophysiological recording

#### ! CAUTION

Follow proper safety precautions when working with and disposing of materials containing human tissues.

**Note:** If enough team members are present and you have both a patch-clamp rig and hdMEA, Option A and Option B can run concurrently.

#### ? TROUBLESHOOTING

If recordings are not yielding activity, or cease to yield activity, repeat Step 20 (TTC staining) to check for slice viability with one of the remaining slices. See Table 4.

### Option A: patch-clamp recording (timing—2–8 h)

**Figure 4:**
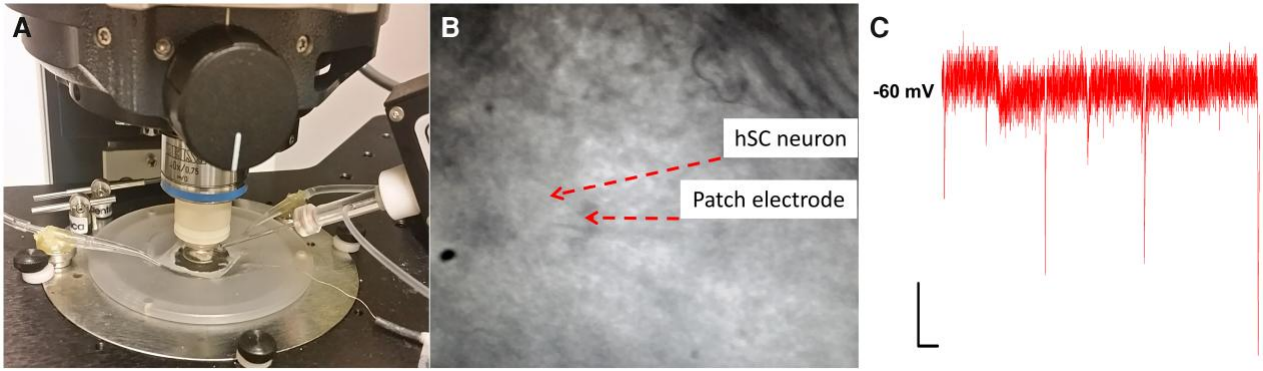
**Patch-clamp recording in human spinal cord slices.** (**A**) A human spinal cord slice is secured with a tissue anchor for recording. (**B**) A patch electrode sealed onto a human superficial posterior horn neuron. (**C**) Representative trace of postsynaptic activity at −60 mV. Scale bars = 1000 ms (*x*-axes), 10 pA (*y*-axes).

*Note: a clear, bright substantia gelatinosa visualized under brightfield optics at low magnification is an indication of a healthy slice. If the substantia gelatinosa is not clear, discard the slice and move to another.

25. Pick up a single slice using the custom dropper. Place it in a small petri dish containing some room temperature, bubbled protective aCSF without kynurenate. Using straight-edge Vannas dissection scissors, cut the slice in half to separate the left/right halves of the hSC. Note, if the slice is small enough in diameter to fit in the microscope well (Fig. 4A) beneath your tissue anchor, or if studying contralateral connectivity, this step can be skipped.
26. Place the hSC hemisection in the immersion chamber, filled with bubbled aCSF, of the patch rig. Secure with tissue anchor, and continuously perfuse with fresh, bubbled solution throughout experiments.
27. Identify your region of interest under brightfield optics. The substantia gelatinosa can serve as a useful marker*. Note that the anatomy of the hSC differs substantially from rodent spinal cord.^69^ Refer to an atlas if needed.^70^

### Option B: hdMEA recording (timing—2–8 h)

28. Depending on the size of the active recording area on your hdMEA, your hSC slice will need to be cut to fit into this area. Our preparation uses a 6 mm × 6 mm recording area; thus hSC cut into one-fourth sections fit well onto the chip. Follow Step 25 above to cut the tissue containing your area of interest to the correct size for your chip.
29. Couple the tissue to the hdMEA chip. Using a P200 pipettor, remove all solution from the chip. Place tissue anchor to secure the slice and immediately pipette on 30 µl aCSF.
30. Remove the aCSF and then immediately replace on the slice, repeating this step three times, thus coupling the slice to the active recording area of the chip.
31. Continuously perfuse at a rate of 1–4 ml/minute with fresh, bubbled solution throughout experiments. Before beginning an experiment, allow the slice to acclimate on the chip for 15 min.

### Timing

**Reagent Setup:** agarose preparation, internal recording solution preparation, slice external recording solution (aCSF): 3.5 h (can be made in advance and kept on hand)

**Reagent setup:** make protective aCSF, cool, dissolve kynurenate, bubble protective aCSF: 2 h

**Equipment Setup:** 1.5 h

**Steps 1–9:** hSC extraction: 30–50 min

**Steps 10–13:** Spinal cord microdissection: 30–60 min

**Steps 14–24:** Tissue sectioning and recovery: 2–4 h

**Steps 25–31:** Electrophysiological recording: 2–8 h

### Troubleshooting

#### Anticipated results

##### Option A: patch-clamp electrophysiology

This protocol can be used to prepare tissue for patch-clamp recording.^35,36^ We patched pain-processing neurons from lamina I of the superficial dorsal horn (SDH; anatomically the superficial posterior horn in humans), which was identified as being within 50 μm medially of tracts that run along the outer edge of the substantia gelatinosa. All slices used had a clear and bright substantia gelatinosa under brightfield optics. Neurons that were selected to be patched had smooth surfaces free of blebs and had lightly defined edges (Fig. 4B).

The overall yield of high-quality whole-cell recordings from adult human spinal cord slices was relatively low, at zero to four high-quality whole-cell recordings per donor with most donors yielding a single suitable cell even when the tissue was healthy. This is consistent with our previous work in patch-clamp recordings of adult rodent spinal cord,^63^ especially when additional pharmacological objectives are being pursued. Several factors contribute to the modest yield. Neuronal visibility is poor in adult human tissue due to extensive myelination, light scattering and the dense neuropil, which makes it more difficult to identify neuronal somata. Technical challenges during patching are also common. Pipette tips can become obstructed by extracellular debris, seals are more difficult to form, and in some cases, it is not possible to break through the membrane even after a high-resistance seal is achieved. In addition, when recording from defined populations of spinal neurons, such as lamina I here, the practical yield can be further limited by the availability of appropriate cells to patch. Although the tissue remained visibly healthy for several hours, it was often challenging to locate neurons in lamina I that were free of debris or passing tracts, had smooth soma surfaces and lacked blebs. Finally, many potential recordings did not meet the stability criteria required for inclusion, such as acceptable access resistance and minimal leak.

The criteria for recording neurons included an access resistance under 30 MΩ and leakage currents no greater than −100 pA at a holding potential (*V*_h_) of −60 mV.^61^ Between voltage-clamp gap-free recordings, a low-amplitude seal test was applied periodically, enabling real-time monitoring of access resistance (R_a_) and membrane capacitance (C_m_) to ensure recording stability. Signals were sampled at 10 kHz and low-pass filtered at 2 kHz. In human lamina I recordings, passive membrane recording properties fell within a physiological range, with R_a_ typically 12–24 MΩ and C_m_ ∼22–84 pF. The liquid junction potential for our internal and external solutions was calculated to be ∼4 mV; given its small magnitude and the fact that our analyses did not depend on determining exact reversal potentials, no online correction was applied.^71,72^

**Figure 5:**
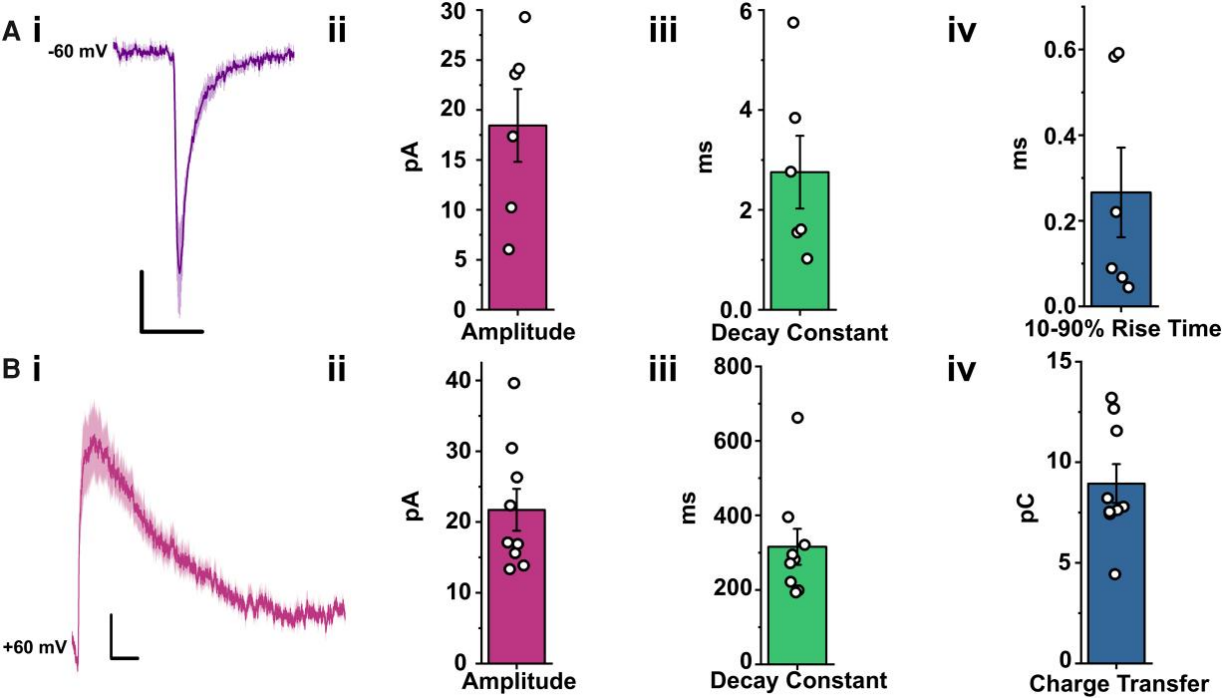
**Whole-cell patch-clamp recording of pharmacologically-isolated mEPSCs at** −**60 and +60 mV.** (**A**, **B**) Averaged mEPSCs of hSC superficial dorsal horn neurons recorded at −60 mV (**A**i, *n* = 6 cells from 4 male and 2 female donors) and at +60 mV (**B**i, *n* = 9 cells, with 7 cells from 5 male and 2 cells from 2 female donors). Recordings were performed in tetrodotoxin (TTX), Cd^2+^, strychnine and bicuculline to isolate AMPAR-mediated inward events at −60 mV and NMDAR-dominated outward events at +60 mV. Scale bars = 10 ms (*x*-axes), 5 pA (*y*-axes). (ii–iv) The amplitude, 10–90% rise time and decay constant of mEPSCs measured from the corresponding traces. Within bar graphs, each data point represents the averaged measurement from a single recorded cell; bars represent the mean, and error bars indicate the standard error of the mean.

Whole-cell patch was established at −60 mV, allowing for the recording α-amino-3-hydroxy-5-methyl-4-isoxazolepropionic acid (AMPA) receptor-mediated miniature excitatory postsynaptic currents (mEPSCs) (Fig. 4C). AMPAR mEPSC responses from six neurons recorded across four male and two female donors show consistent waveform characteristics (Fig. 5Ai). Measuring the average amplitude (18.44 ± 8.93 pA), 10–90% rise time (0.27 ± 0.26 ms) and decay constants (2.76 ± 1.78 ms) across the sampled neurons provided insights into the biophysical properties of these excitatory synaptic responses in the human SDH at −60 mV (Fig. 5Aii–iv), with faster activation and deactivation kinetics compared to that previously observed for rat lamina I neurons.^61^

The holding potential was then adjusted from −60 to +60 mV to record NMDAR-dominated mEPSCs. All +60 mV recordings were performed in the presence of tetrodotoxin (TTX), Cd^2+^, strychnine and bicuculline to block action potential-dependent release and eliminate glycinergic and GABA_A_ receptor-mediated currents. To ensure that only NMDAR-mediated synaptic charge was quantified, analysis was restricted to a window beginning 40 ms after mEPSC onset, which excludes the fast AMPAR component and isolates the slow NMDAR-mediated decay. Under these conditions, the outward events primarily reflect NMDAR-dominated miniature currents, consistent with prior characterization in adult lamina I neurons,^61^ Fig. 5Bi illustrates averaged mEPSCs (*n* = 9 cells) from five male and two female donors, displaying decay constants (315.61 ± 144.66 ms), charge transfer values (8.99 ± 2.90 pC) and amplitude measurements (21.72 ± 8.90 pA; *N* = 9) of saline-treated neurons (Fig. 5Bii–iv). These experiments characterized the biophysical properties of NMDAR-mediated mEPSCs, which is important for understanding the kinetic profile and synaptic function of these currents in human SDH neurons. This foundational knowledge is critical for linking specific synaptic properties to functional outcomes.

#### Option B: hdMEA recordings

**Figure 6:**
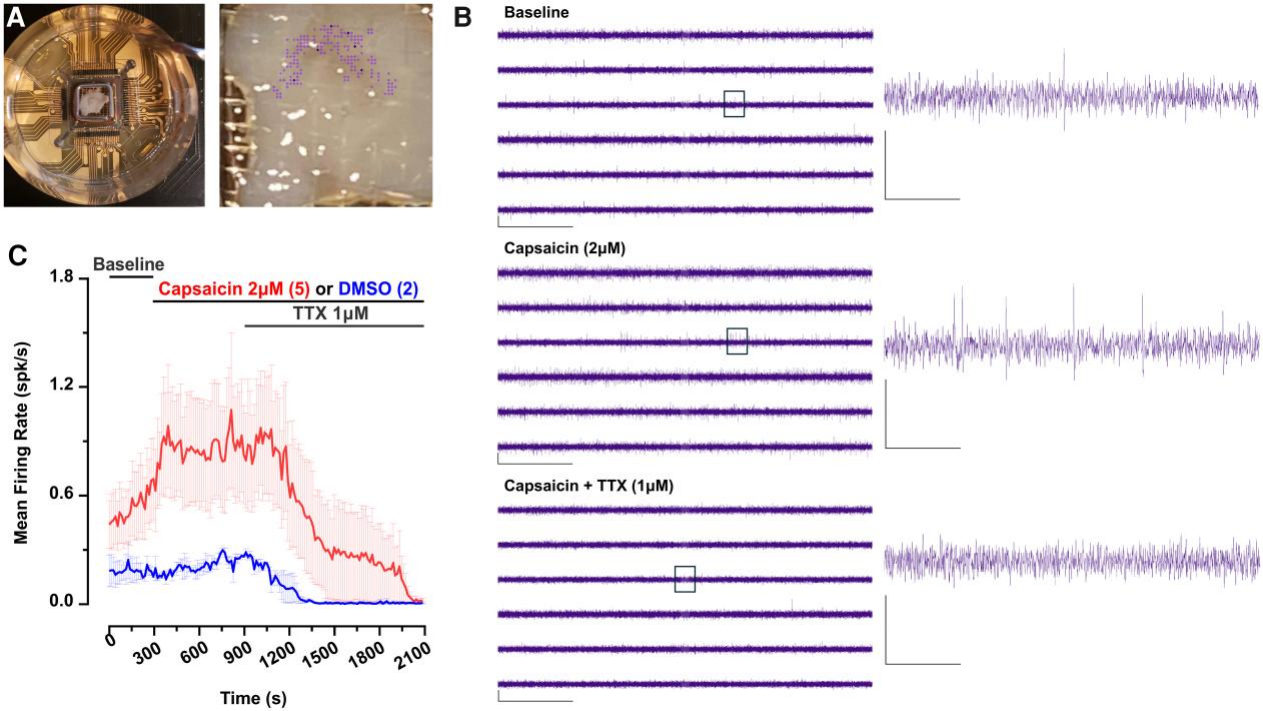
**High-density multielectrode array (hdMEA) recordings reveal spontaneous and pharmacologically evoked spiking in hSC neurons.** (**A**) A human spinal cord slice dorsal horn quadrisection mounted on the hdMEA chip, secured by a tissue anchor *(left)*, with active electrodes in the superficial dorsal horn (SDH) indicated in purple *(right).* (**B**) Representative traces from selected SDH electrodes (indicated with dark purple in **A**) during baseline *(top)*, application of 2 μM capsaicin *(middle)* and subsequent co-administration of 1 μM tetrodotoxin (TTX) *(bottom)*. Scale bars: *x*-axis =1 s, *y*-axis = 50 µV. *Right:* zoomed-in view of representative spikes recorded at the same time intervals. Scale bars: *x*-axis =100 ms, *y*-axis = 50 µV. (**C**) Mean firing rate of putative SDH neurons during baseline, capsaicin and TTX conditions. Capsaicin recordings: *n* = 5; DMSO vehicle: *n* = 2; recordings obtained from *n* = 2 donors (1 male, 1 female).

High-density multi-electrode array (hdMEA) recordings are valuable for studying the electrophysiological properties of populations of neurons in an intact network, allowing for the high-resolution detection of neuronal activity and comprehensive analysis of spike properties across multiple sites. Here, we demonstrate the utility of hdMEAs for recording from hSC slices. Recordings were made using 3Brain Accura 6 mm CMOS HD-MEA chips containing 4096 electrodes arranged in a 64 × 64 grid with 60 μm inter-electrode spacing and ∼20 μm electrode diameter; signals were sampled at 20 kHz per channel, band-pass filtered at 300–3000 Hz and acquired using the system’s built-in amplification. In Fig. 6A, we show a quadrisected hSC slice mounted on the hdMEA chip, secured with a tissue anchor to ensure stable recordings. Active electrodes within the SDH are shown in purple. Representative traces from selected electrodes (Fig. 6B, left) illustrate baseline spontaneous activity, the increase in firing following application of 2 μM capsaicin and the subsequent suppression of activity by 1 μM TTX. A zoomed-in view of an individual channel recorded across adjacent electrodes is on the right (Fig. 6B, right).

Using the hdMEA system for pharmacological experiments further demonstrates the versatility of hdMEA recordings for investigating dynamic changes in neuronal activity. Capsaicin robustly increased firing rates in SDH neurons (*n* = 5 recordings), whereas the DMSO vehicle did not alter activity (*n* = 2 recordings) compared to baseline. Application of TTX abolished nearly all spiking, confirming that the recorded events were physiological, sodium channel-dependent action potentials (Fig. 6C). The experiments, conducted using slices from two donors (one male, one female), illustrate the potential of hdMEAs for testing modulators like BDNF, which are known to drive hyperexcitability in male nociceptive networks of the hSC.^36,37^

Overall, these tools provide a robust platform for exploring the impact of various agents on human neuronal excitability. This capability is critical for identifying compounds that may inhibit pathological excitability and understanding mechanisms that exacerbate excitability, shedding light on pathways and therapeutic targets relevant to pain and other neurological disorders of the human spinal cord.

## Supplementary Material

fcag157_Supplementary_Data

## Data Availability

Data sharing is not applicable to this article as no new data were created or analysed in this study.
