# Supplementary material for: Processing and sectioning of organ donor spinal cord tissue for electrophysiology on acute human spinal cord slices
Source: Brain Commun. 2026 Apr 29;8(3):fcag157. doi: 10.1093/braincomms/fcag157 (PMC13179502; doi:10.1093/braincomms/fcag157)
Supplement: fcag157_Supplementary_Data [file fcag157_supplementary_data.zip › Supplementary_Materials.pdf]

Supplementary Materials

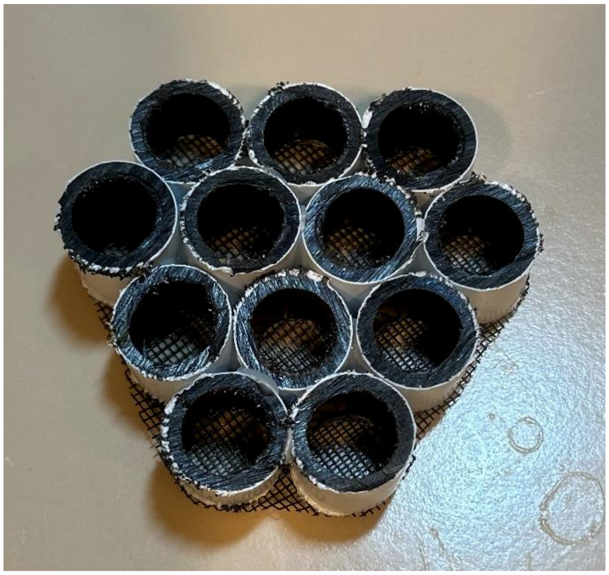

Supplementary Figure 1. Custom slice recovery chamber. This slice recovery chamber is made of 1" PVC piping glued onto a plastic screen. This size, with 12 individual chambers, fits into a 1L beaker. A smaller slice chamber is used to allow slices to passively to room temperature and contains 9 individual chambers.
