# Supplementary material for: Processing and sectioning of organ donor spinal cord tissue for electrophysiology on acute human spinal cord slices
Source: Brain Commun. 2026 Apr 29;8(3):fcag157. doi: 10.1093/braincomms/fcag157 (PMC13179502; doi:10.1093/braincomms/fcag157)
Supplement: fcag157_Supplementary_Data [file fcag157_supplementary_data.zip › Supplementary Video Caption .docx]

**Supplementary Video: Human spinal cord tissue dissection and sectioning.**

Video illustrating the removal of meninges and vascularization from a human spinal cord tissue segment under a dissecting microscope. Following this, thin transverse spinal cord sections are cut using a vibratome and then transferred to a bath for trimming and recovery.
